# Hospital-to-home transitions of patients with lung cancer – a qualitative study of primary healthcare professionals’ experiences

**DOI:** 10.1186/s12912-026-04638-4

**Published:** 2026-04-10

**Authors:** Charlotte Nilsen, Gro Beate Samdal, Sidsel Ellingsen

**Affiliations:** 1 Centre of Diaconia and Professional Practice, VID Specialized University, Bergen, Norway; 2 Faculty of Health Sciences, VID Specialized University, Bergen, Norway

**Keywords:** Healthcare professionals, Lung cancer, Primary care, Qualitative research, Hospital-to-home transition

## Abstract

**Background:**

Patients with lung cancer are considered a vulnerable patient group. Often with a high symptom burden, complex needs, and are frequently transferred between hospital and home, which can demand considerable resources from healthcare professionals. Our study aims to explore how general practitioners and registered nurses working in home care experience hospital-to-home transitions of patients with lung cancer.

**Methods:**

Descriptive qualitative design. Five GPs and 16 RNs working in home care services in three Norwegian municipalities were included, distributed across three focus group interviews, two dyadic interviews, and one in-depth interview. Data was analysed using an inductive thematic approach.

**Results:**

Registered nurses and general practitioners faced challenges collaborating with specialised healthcare providers during hospital-to-home transitions, negatively affecting patients’ home healthcare. They reported that healthcare professionals in specialised care lacked an understanding of primary healthcare services, resulting in a deficiency of health information for continued care at home. Registered nurses highlighted an approach to at-home care that differs from specialised healthcare, prioritising quality of life and patient preferences over the diagnosis itself. Additionally, delayed information flow deprived general practitioners and registered nurses of crucial information. When a patient’s condition deteriorated, the lack of medical support from general practitioners often caused registered nurses to hospitalise patients for appropriate medical treatment.

**Conclusion:**

The findings reveals that primary and specialised care professionals operate within different ‘logics of care’, leading them to prioritise differently and hindering cooperation in hospital-to-home transitions. Enhancing communication and collaboration between specialised and primary care is essential for high-quality hospital-to-home transitions of patients with lung cancer. Establishing more frequent face-to-face meetings and interdisciplinary team discussions can facilitate greater mutual understanding of patients’ needs among healthcare professionals in primary and specialised care.

## Introduction

Lung cancer was the most frequently diagnosed cancer globally in 2022, with almost 2.5 million new cases, and is the leading cause of cancer-related death, with about 1.8 million deaths annually [1]. In most countries, the 5-year survival rate for patients with lung cancer is below 20%, and many are diagnosed with lung cancer at a late stage, leaving life-prolonging and palliative therapy as the only options. However, the 5-year survival rate has increased in many countries due to increased preventive screening, targeted treatment, and better follow-up. Therefore, more patients are now living longer with lung cancer [1], highlighting the importance of focusing on better care pathways for patients with lung cancer across the different levels of the healthcare system.

Caring for vulnerable patients, such as those with low socioeconomic status and low health literacy, older patients, and patients with multimorbidity, carries a greater risk of harm [2, 3]. Patients with lung cancer exhibit all these vulnerability characteristics [4–7]. They report a lower quality of life than patients with other types of cancer and a high rate of symptom burden, such as distress due to respiratory symptoms, fatigue, pain, and sleep disturbance [8–11].

In 2012, the Norwegian Coordination Reform was implemented to provide a better care pathway for patients receiving specialised and primary care services [12]. This reform transferred more responsibility from specialised care to primary care, aiming to reduce the number of hospitals stays. Specialised care has the authority to determine when a patient is medically ready for discharge and can be discharged, at which point primary care assumes responsibility for the patient’s ongoing management, with support from specialised outpatient services as required. Patients with cancer, including lung cancer, now receive more outpatient treatment at specialised care clinics while continuing to live at home [13]. When required, patients are cared for by the local home-based care office between treatments at hospital-based specialised care clinics. As a result, patients with lung cancer are transferred between the hospital and their home more frequently, and healthcare professionals (HCPs) in primary care now care for a growing number of patients receiving specialised treatment.

The hospital-to-home transition is a vulnerable stage in a patient’s trajectory [14–17]. The length of hospitalisation after lung cancer surgery has also been radically reduced [10], and research shows that hospital-to-home transitions after surgery can be challenging for patients with lung cancer; they may feel insecure, inadequately informed, or unprepared for a hospital-to-home transition that proves more challenging than anticipated [16, 18, 19].

In Norway, when patients are transferred from the hospital to their home, registered nurses (RNs) and general practitioners (GPs) in primary care continue the care initiated by HCPs in specialised care, relying on the hospital’s cooperation and documentation. However, research indicates that RNs providing home-based care express concerns about inadequate communication with HCPs in specialised care, dissatisfaction with service coordination during hospital-to-home transitions, and the general quality of those transitions [20, 21]. To continue the care initiated by HCPs in specialised care and to provide palliative care when required, RNs providing home care must be able to consult a physician, usually the patient’s GP. However, according to Norwegian statistics, few patients receive end-of-life care at home provided by their GP due to a lack of resources. Kjellstadli et al. [22], and Skinner et al. [23] revealed that municipal RNs rank collaboration with GPs as the worst in horizontal collaboration in primary care services.

Caring for patients with lung cancer in hospital-to-home transitions can demand considerable resources from HCPs. This study is part of a larger project exploring hospital-to-home transitions for patients with lung cancer in Norway. While the first study focused on the experiences of physicians and RNs in specialised care [24], this study examines the experiences of GPs and RNs in home-based care. Little knowledge exists on how HCPs in primary care experience caring for patients with lung cancer in hospital-to-home transitions, and how RNs providing home-based care and local GPs cooperate to ensure quality in transitions and throughout the care continuum. In order to help close this research gap, this study aims to explore how RNs and GPs experience hospital-to-home transitions for patients with lung cancer. In this article, we also aim to discuss how the broader healthcare system facilitates primary care services and enables RNs providing home-based care and local GPs to maintain their interprofessional collaboration.

## Materials and methods

In the start-up phase of our study, a reference group consisting of a local GP, an RN working at a home-based care office, a physician specialising in lung diseases, an RN working in a hospital inpatient ward (pulmonary unit), an RN working in an outpatient clinic (pulmonary unit), and a patient representative from the Norwegian Cancer Society provided different perspectives on our study’s purpose, aim, design, and interview guide. The first author also met with the patient representative to gain insights into his experience with hospital-to-home transitions.

### Study design

A descriptive, qualitative design with an open, flexible, and exploratory approach was used to capture nuances and variations in the participants’ experiences [25].

### Setting

Our study was conducted in primary care settings within one urban (285,000 inhabitants) and two rural (1,600 and 38,000 inhabitants) municipalities in Norway. Its focus was on home-based care provided by municipal home-based nursing care services and general medical services provided by local GP clinics. Most Norwegian citizens are enrolled with a GP through the national healthcare system [26].

Healthcare in Norway is universal, funded by a fully funded healthcare system, where patients pay only a minor user fee for services. Paid user fees are registered and count toward the accumulation of an exemption card for public health services. Specialised care services are organised through four state-owned regional health enterprises, ensuring the population has free access to specialised care through hospital admission or outpatient clinics.

### Participants

The inclusion criteria were (a) working as an RN in a home-based care office or as a GP at a local GP office and (b) having experience with hospital-to-home transitions for patients with lung cancer, including experience with those who had received curative treatment, outpatient consultations, palliative treatment, and no treatment.

Purposive sampling was used to include participants [25]. The GP from the reference group assisted in recruiting local GPs as study participants, and the RN working at a home-based care office from the reference group contacted the leaders at home-based care offices, who acted as gatekeepers and recruited RNs for the focus groups. No information was collected if a prospective participant refused to participate. The gatekeepers determined the composition of the focus groups, explaining their varying composition. The researchers had no prior contact with the participants before the interviews but exchanged emails with the GPs to schedule interviews.

### Data collection

Data were collected through three semi-structured focus group interviews with RNs providing home-based care, two semi-structured dyadic interviews with GPs, and one semi-structured individual interview with a GP. The focus group interviews with RNs were held at their home care office, whereas the dyadic and individual interviews with GPs took place in their local clinics. Data collection occurred from March to June 2021.

The authors developed a semi-structured interview guide with 11 questions (Table 1). The participants were asked to focus their answers on their experiences with patients with lung cancer. Six interviews (Table 2) were conducted, each lasting approximately one hour, and were recorded using a tape recorder. The first author led the interviews, while the third author and a research colleague served as co-moderators. The sample size was considered sufficient to answer the research question.

**Table 1:** The semi-structured interview guide used for the three focus group interviews, two dyadic interviews, and one in-depth interview

	Question	
1	Presentation round: Please tell us a little about yourself. How long have you worked as an RN in home-based care, and what are your work tasks? (Introductory questions/icebreaker)	
2	What sort of treatment and care do you provide for patients with lung cancer? - Is there anything special about patients with lung cancer or about working with them? If so, please explain.	
3	Could you please walk me through what you do for patients with lung cancer at hospital-to-home transitions? - What resources do you need to provide good patient care during a hospital-to-home transition?	
4	What knowledge does the patient need when transferred to their homes? - What information is most important for patients to understand before being transferred to their homes?	
5	*Questions for RNs*: Could you tell me how you provide information to the patient after the hospital-to-home transition? - How do you determine whether the patient understands and remembers the information provided? - How do you involve the patients’ relatives when you provide information?	*Questions for GPs*: Could you tell me how you provide information to the patient when you meet them at consultations after the hospital-to-home transition and what follow-up you provide? - How do you determine whether the patient understands and remembers the information provided? - How do you involve the patients’ relatives when you provide information?
6	Could you share some examples of individual adaptations provided for the patients?	
7	How is the patient involved in planning their future after the hospital-to-home transition?	
8	*Questions for RNs*: How do you cooperate with the hospital healthcare professionals in hospital-to-home transitions, and how do you communicate with them?	*Questions for GPs*: How do you cooperate with the hospital healthcare professionals in hospital-to-home transitions, and how do you communicate with them? - How are you involved in the patient’s treatment trajectory?
9	How should the patient’s home be organised and prepared before discharge? - Who is responsible for preparing the patient’s home?	
10	Is there anything we have not discussed that you consider essential for patients with lung cancer in hospital-to-home transitions?	
11	What do you believe is the most important topic discussed today?	

**Table 2:** Characteristics of the participants in the three focus group interviews, two dyadic interviews, and one in-depth interview recruited from March to June 2021 (*N* = 21)

Interviews (*n* = 6)	Type of interview	Professional background (participant ID)	Sex	Setting
1	Dyadic	General practitioner (GP1, GP2)	2 females	GP office, urban area
2	Focus group	Three registered nurses and one cancer coordinator (N1, N2, N3, N4)	4 females	Home-based care office, rural area
3	Focus group	Registered nurses (N1, N2, N3, N4)	4 females	Home-based care office, urban area
4	In-depth	General practitioner (GP)	1 male	GP office, urban area
5	Focus group	RNs (N1, N2, N3, N4, N5, N6, N7, N8)	7 females, 1 male	Home=based care office, urban area
6	Dyadic	General practitioner (GP1, GP2)	2 males	GP office, rural area

### Data analysis

The first author transcribed the interviews verbatim, and the transcripts were not returned to the participants for commentary. Data were analysed using a six-step inductive thematic process inspired by Braun and Clarke [27–29] (Table 3). While the data consisted of focus group interviews, dyadic interviews, and an individual interview, it was analysed as a whole. The same interview guide was used in all interviews, except for a few questions adapted to the practice of RNs or GPs. Analysing the material as a whole helped identify overarching themes and trends that cut across the different data sources, which was the intention of using a thematic analysis.

**Table 3:** Establishing trustworthiness during each phase of the thematic analysis

Phase of the thematic analysis	Means of establishing trustworthiness
Phase 1: Familiarisation with the data	First, all authors read all interviews several times, familiarising themselves with the data and noting ideas for themes for further analysis. This process was followed by a discussion of ideas and possible themes.
Phase 2: Generating initial codes	In the second step, the first and last authors generated initial codes across the entire data corpus using the qualitative computer software package NVivo [30]. Altogether, the data was analysed into 44 semantic codes.
Phase 3: Searching for themes	In the third step of the analysis, the first and last authors used a visual board to visualise code relationships, which developed into nine possible introductory themes.
Phase 4: Reviewing themes	In the fourth step, the first author reviewed the nine themes related to the initial nine codes, going back and forth between themes, codes, and the entire data corpus. Finally, a thematic map with two themes was identified.
Phase 5: Defining and naming themes	In the fifth step, a thorough analysis was written for each theme, and sub-themes were identified for each theme.
Phase 6: Producing the report	In the last step of the analysis, the full report was written, and examples from the interviews were extracted to demonstrate and capture the essence of themes and sub-themes. Finally, all authors read the full report and jointly conducted an additional review of the themes to capture their essence.

### Ethical considerations

The Regional Committee for Medical and Health Research Ethics (REK Western Norway) assessed that the project was not subject to review according to the Norwegian Health Research Act (reference number 240604). The Norwegian Agency for Shared Services in Education and Research (Sikt) approved the processing of confidential data (reference number 190602). Fieldwork permission was obtained from all participating municipalities. According to national regulations, no further ethics approval was required. All participants provided informed consent to take part in our study. The interview transcripts and audiotapes were kept in separate locked files that could only be accessed by the authors.

### Trustworthiness

In order to assure credibility, the Standards for Reporting Qualitative Research (SRQR) were used to enhance quality and transparency [31]. The reference group helped ensure our study’s relevance from a clinical perspective. Dependability was ensured by having all researchers participate in the analysis, which was performed step-by-step, continuously going back and forth between the interviews to ensure that every aspect was included. The three authors are all RNs but have different backgrounds in the use of theory, methods, and research areas, which was considered a strength throughout the process and in assuring confirmability. Finally, authenticity was ensured by providing rich citations from the interviews throughout the results, demonstrating the varied discussions among the participants.

## Results

### Participants

Five GPs and 16 RNs working in home care services in three Norwegian municipalities were included.

All participating GPs were specialists in general medicine with experience ranging from 18 to 30 years. Some of the participating RNs had undertaken continuous professional education in oncology. The participating RNs’ experience in providing home-based care varied from 6 months to 30 years; 10 RNs had ≥ 8 years of experience. A local cancer coordinator (2-N3–2 represents the group number and N3 represents the participant’s ID) also participated in one of the focus group interviews; she was also an RN. In Norway, a cancer coordinator is an HCP who coordinates and facilitates cancer care services within a specific geographic area or region.

The analysis revealed that all participants experienced hospital-to-home transitions as challenging in general, but especially for patients with lung cancer, which they viewed as a vulnerable patient group often needing extra resources at home compared to other cancer patients. Two overarching themes, each with two sub themes each were identified (Table 4).

**Table 4:** The themes and sub-themes identified in three focus group interviews, two dyadic interviews, and one in-depth interview through inductive thematic analysis

Theme	Sub-themes
A need for better collaboration between primary and specialised care.	Specialised care lacks an understanding of primary care
	Deficient information from specialised care
A need to focus on patients’ quality of life while navigating across barriers.	Emphasise patients’ quality of life, not just their treatment
	Lack of professional cooperation within primary care

### A need for better collaboration between primary and specialised care

RNs and GPs both perceived that HCPs in specialised care did not always understand the content and limits of primary care services, nor the extra challenges they face when caring for patients at home.

#### Specialised care lacks an understanding of primary care

The RNs and GPs expressed that HCPs in specialised care made promises to patients that the RNs providing home-based care could not fulfil. The participants expressed an understanding that HCPs in specialised care are under the same pressures regarding efficiency and lack of resources. However, they called for a greater understanding of the limitations in their home-based care setting, as illustrated by the following discussion among RNs:

*3-N1: Based on what the patients are saying and what’s written in the nursing summary*,* it seems that they [HCPs in specialised care] talk about home care nursing as an everlasting resource that can show up at any time. Because some of them [patients] seem to expect us to move in with them. … It seems as if the hospital brags about us to make the patient feel safe about going home. And then*,* when the patients returned to their homes*,* it was not quite what they had imagined.*

*3-N4: They [patients] get the wrong expectations.*

*3-N3: I simply don’t think everybody knows what home care nursing is.*

*3-N4: Quite a few things are promised on our behalf.*

*3-N1: Yes. They might have promised that we will come at a specific time in the morning and how many times each day. But strictly speaking*,* it is not for them to decide.*

As a result of how HCPs in specialised care portrayed home care to patients with lung cancer, some patients imagined that everything would improve once they returned home. Still, often, it was more challenging than anticipated:

*3-N3: They’re suddenly alone*,* you know. It is totally different*,* a new everyday life.*

*3-N1: Yeah*,* you are all alone*,* and then you have problems with your breathing*,* and it is hard to get around*,* and you get scared.*

*3-N2: It is two completely different situations*,* you know. They come from hospitals where everything is facilitated and where they have people around them all the time*,* and then they come home*,* and then it’s not like that anymore.*

*3-N1: And although we [the RNs] are close*,* we’re still far away. They do not have the security that we will come running. … We might be several kilometres away.*

The RNs explained that when patients returned from the hospital, their total care needs were assessed by RNs providing home-based care. The information received from the HCPs in specialised care was often inadequate, missing essential details relating to the patient’s everyday life at home:

*3-N4: It is hard to visualise at the hospital what they need at home. You kind of think*,* ‘Oh*,* they need a walker at home’*,* but you don’t think about the fact that the house is built so that the walker cannot be used … that the threshold to the bathroom is too high.*

According to the RNs and GPs, it had been a common practice to hold collaborative meetings with patients with lung cancer, their relatives, their GPs, RNs in home-based care, and HCPs in specialised care before hospital-to-home transitions if they have special needs, such as advanced palliative care. Lately, such collaborative meetings have been phased out. Now, HCPs in specialised and primary care communicate and plan hospital-to-home transitions via electronic messages. However, with this new practice, important information is often lost in that intermediate stage or arrives too late, making it difficult to plan. An experienced RN described the previous practice as follows:

*5-N2*: [In times past] *everybody sat around the table*,* and everybody received a task: ‘You do this*,* and you do that’. So*,* when the patient came home*,* everything was arranged in advance. Now a* [electronic] *message is sent out*,* then we get a patient home*,* and what often is the problem is that we don’t know how ill the patient really is. I mean*,* will they live for four days*,* or will they live for six months? You know*,* we never know how ill they are or how many resources they need before we have seen the patient.*

The GPs felt that, after the collaborative meetings had been phased out, they had no say in what responsibility they were given in the patient’s trajectory. In addition, their number of tasks had increased after the Coordination Reform without a corresponding increase in resources allocated to primary care. Two GPs expressed their frustration as follows:

*1-GP1: It* [the responsibility] *is constantly being pushed over to the GPs. They* [the patients] *now leave the hospital sooner. Therefore*,* more checkups must take place at the GP’s office.*

*1-GP2: It was pretty extreme for a period. It’s gotten better. It was as if the GP was a mercantile service.* [Citing a discharge summary example: ] *‘It was not possible to take blood samples today. Ask the GP to take the blood samples.’ That is using us as secretaries.*

#### Deficient information from specialised care

The GPs expressed frustration that the physicians’ discharge summaries after inpatient or outpatient consultations were not more specific about which follow-up procedures were their responsibility. For instance, they felt uncomfortable when patients saw them for checkups without the entire treatment plan or, worse, when they had not received the inpatient or outpatient clinic discharge summary. The RNs said they often did not receive important information after the patient’s consultations at the hospital’s outpatient clinic. Typically, the physicians sent outpatient discharge summaries to the patient’s GP, but the RNs rarely received copies. If the RNs providing home-based care had questions or needed to forward information about the patient to the outpatient clinic, they had to make contact by phone. Due to the lack of communication between RNs in specialised and home care, the patients sometimes had to supply the information themselves.

*2-N3: It’s a big problem that [RNs at] the outpatient clinic can’t send us electronic messages because a lot [of information] is lost in the follow-up. And we have no idea what they’re doing [at the hospital] … we are not notified of changes. Or when we visit them [at home] to give medications*,* they may have received a distressing message [at the outpatient clinic] that day*,* and we have no information on what they have been told … And when they get home*,* they ask us*,* and we don’t know anything. It’s terribly uncomfortable.*

*2-N4: More and more patients receive their treatment in outpatient clinics.*

*2-N3: Yes*,* many do*,* especially lung cancer patients.*

*2-N4: Yes*,* and previously*,* we didn’t receive the outpatient discharge summary [from the physician]*,* but at least we do now. But we usually receive it some days later.*

*2-N3: You receive a message*,* but there is no room for dialogue … And sometimes they [hospital physicians] initiate medical injections for the patients. Then we go to the patient to do that*,* and we barely know their diagnosis. The hospital might think that we don’t need that information … but when we get to their house*,* they ask us. They might have nausea and pain*,* and we barely know the reason.*

Because RNs providing home-based care see the patients in their everyday environments, they observe challenges not perceived at the outpatient clinic. Without a way of communicating through electronic messages, essential issues about the patient could be overlooked; this was especially critical if a patient had difficulty remembering or did not have sufficient support from relatives, as one nurse stated:

*5-N6: The patient can appear mentally clear and able to answer questions when meeting the hospital’s HCPs. But then it turns out that the patient is not*,* and that is not discovered at the hospital. The patient can say that everything is going great at home*,* but that is not the reality we see in their homes. But we don’t communicate with the physician at the hospital*,* and we are not asked either.*

Furthermore, the RNs and GPs emphasised the importance of having a shared understanding of the treatment plan and prognosis with patients and their relatives. They expressed that, in their experience, as long as the patients were treated at the hospital, the hospital retained most of the responsibility through frequent appointments at the outpatient clinic. They also reported that the information provided was often fragmented. Only when all treatment ended and they assumed responsibility for end-of-life care were they provided with more information. Regardless, as long as they were involved as healthcare providers, they called for extended information throughout the entire trajectory. Patient information, they advised, should be provided both orally and in writing before discharge, along with whom to contact at different hours. Without such measures, challenging situations were likely to arise, as one RN exemplified:

*5-N6: Once*,* we received an electronic dialogue message* [from the hospital] *of possible metastasising to the bones in a lung cancer patient. And in this case*,* neither the patient nor the relatives were informed. And then it was briefly mentioned by us. He had so much pain*,* and the order was to help the patient with pain management* [due to suspected metastasising]. *It almost led to a family disaster when we implied that it might be due to metastasising to the bones. Then*,* we received feedback from the patient’s GP that nothing was confirmed. In the end*,* it turned out that that* [metastasising to the bones] *was the explanation for the pain. But when these things are not clarified*,* it becomes challenging. … We feel guilty because we had said something we shouldn’t have said. We were yelled at for saying what we did.*

Access to the proper medications was identified as a significant challenge in hospital-to-home transitions. Hospital physicians typically prescribe medications for the patient to be continued at home. The patient or their relatives are asked to collect the prescriptions from the pharmacy after discharge. However, medications prescribed for lung cancer treatment are often rare and difficult to obtain from the local pharmacies. Since RNs providing home-based care are often responsible for managing patients’ medications, they assume responsibility for collecting the prescriptions from the pharmacy. The RNs reported how this led to additional work, prompting them to double-check all information and inclining them to distrust the information they received:

*3-N4: Relatives often get frustrated because they often don’t have the medications at our local pharmacy. So*,* relatives have to drive around town to get it. And if they don’t have relatives*,* we* [RNs] *must drive to the hospital pharmacy to get the medications. There is so much extra work for these simple things that should be in place.*

*3-N1: Things that should have been done.*

*3-N4: We spend much time on this instead of getting to know the patients … And they* [hospitals] *have checklists that they use to discharge patients*,* but somehow*,* they go like*,* ‘Oh no’*,* and then they forgot that prescription.*

*3-N2: Precisely, that is something we spend an enormous amount of time on.*

*3-N4: The recent trend is them* [hospitals] *sending us the wrong list of medications or an outdated list.*

### A need to focus on patients’ quality of life while navigating across barriers

All participants spoke enthusiastically about how they focus on the general well-being of patients with lung cancer, not just their diagnosis and treatment. They discussed how they provided care based on how the patients wanted to live their lives and took great pride in this aspect of their work. However, the RNs experienced challenges in providing sufficient care at home due to a shortage of medical support from GPs.

#### Emphasise patients’ quality of life, not just their treatment

When the RNs and GPs spoke about how they cared for patients with lung cancer, they emphasised a responsibility beyond treating the lung cancer diagnosis. They focused on the patients’ resources and quality of life and how to assist them in living the best possible life alongside the challenges of the disease.

The RNs always had an initial conversation with new patients, focusing on getting to know them and providing the necessary information. Sometimes, the patient’s needs could differ from what the RNs had initially presumed based on the hospital’s documentation. In addition, when asked about how they cared for patients with lung cancer, the RNs immediately thought of palliative care, explaining that most of their experience with patients with lung cancer involved palliative and end-of-life care. However, they acknowledged that patients with lung cancer were often especially vulnerable and could require more resources than patients with other cancers. Moreover, they described that they perceived patients with cancer as a group that often needed a ‘care package’, regardless of the cancer type:

*5-N2: I mean*,* we don’t really do anything special* [with patients with lung cancer]. *We focus on the overall picture. … Of course*,* it’s the breathing*,* bed rest*,* and these sorts of things. But we see them* [patients with lung cancer] *as palliative patients. There is no difference whether it is lung cancer or a different type of cancer.*

The GPs stated that they recognised their patient from previous appointments. This familiarity allowed them to address additional factors essential for enhancing the patient’s quality of life, as one GP exemplified:

*6-GP1: We followed our patients for several years in a life course that proceeded in different directions. Then a patient turns 85* [years], *gets lung cancer*,* and maybe has other diseases*,* but now that* [the lung cancer] *is the focus. We may have followed them and their family*,* who may also be our patients. Lung cancer will often involve many people on our* [patient] *list. Children*,* grandchildren*,* and partners may also talk about this when they come to me for consultations.*

#### A lack of professional cooperation within primary care

Good cooperation between the RNs and GPs is required to ensure that the responsibility placed on primary care in a hospital-to-home transition is fulfilled. However, the participants described significant challenges that impeded them from doing so. The local home-based offices in larger municipalities cooperate with numerous GPs. All GPs participating in our study had nothing but praise for the work of the RNs providing home-based care, and they described the collaboration with them through electronic messages as efficient and valuable. However, the RNs experienced that GPs’ follow-up of patients varied, such as whether they performed home visits to patients who were too ill to visit their office. In order to care for terminally ill patients, the RNs needed a physician to consult and, if necessary, prescribe new treatments. Without medical support, they had to send terminally ill patients by ambulance to the emergency room, where they were often admitted to the hospital, where they spent their final days. Some RNs described the collaboration with GPs as follows:

*3-N1: Some GPs are hands-on*,* while others are a little more distant*,* presuming it* [responsibility] *belongs to the palliative care team.*

*3-N2: Some* [patients] *don’t know their GP at all*,* or they* [the GP] *do not know them.*

*3-N4: Very challenging. Because we have a crazy number of GPs to deal with. I mean*,* it’s almost every GP in the municipality. What is it*,* 300 or something?*

*3-N1: Some perform home visits*,* and others want to avoid home visits. Some are very hands-on and go* [to the patient’s] *home and join us in planning. Are active in the meeting.*

*3-N4: Some do like this* [covers her eyes with her hands]. *They are hiding*,* and don’t answer our messages.*

The RNs had experience with terminally ill patients with lung cancer who were transferred from the hospital to spend their last days of life at home. However, many GPs lacked this expertise in palliative care. As mentioned, the RNs took great pride in caring for terminally ill patients who wished to die at home, but this could only be provided in cooperation with a physician:

*5-N2: […] I can only speak for myself in what I need when I take care of these patients*,* that is*,* to receive help if I need help. Because we should feel safe when we are in that situation. If we don’t feel safe with patients*,* this will transmit to the person who is dying or is very ill. So* [we need] *a place where we can get help. … We are often very alone in home-based care; we are left to ourselves. I mean*,* we are not in an inpatient ward. We can’t walk out into the hallway and fetch a physician like they can at the hospitals. So having someone to contact when a patient’s condition worsens*,* I think that is very*,* very important for my own sake. You feel very helpless when you are at home with a patient who is about to die*,* and you don’t know what medication to give them.*

## Discussion

Our results provide insights into the experiences of GPs and RNs in primary care during hospital-to-home transitions for patients with lung cancer, addressing what HCPs need to provide adequate care and treatment to patients at home. Specifically, our results show how HCPs in primary care prioritise patient-centred care in transitions, where the patient is seen as ‘more than a diagnosis’. One central finding is that RNs and GPs in primary care are challenged by the lack of understanding by HCPs in specialised care of their needs, resources, and practices. This lack of understanding impedes continued professional care and treatment at home after hospitalisation or outpatient consultations. Here, we argue that these challenges indicate different ‘logics of care’ [32] in primary and specialised care, which are in many ways incompatible. Furthermore, we argue that such misalignment may undermine the practice of hospital-to-home transition.

### Logics of care

Annemarie Mol [32] theorised about the characteristics of a ‘logic of care’, in which logic is seen as embedded in healthcare practices and guiding HCPs’ everyday performances on how to act and react in certain situations. However, the logic of care is neither an official policy nor a tangible guideline. Those encapsulated by a logic of care are not necessarily aware of its characteristics; instead, it is embedded in the practices as ‘common sense’ [32] and, thus, largely taken for granted. As our findings demonstrate, RNs and GPs in primary care exhibit such a shared understanding, or logic of care, connected first and foremost to their focus on home care for patients with lung cancer and how to ensure their quality of life.

In our study, RNs reported that essential patient information was often not transferred to them from specialised care. This finding is consistent with a study by Nilsen et al. [24], who showed how HCPs in specialised care struggle to transfer responsibility to primary care because they fear that patients with lung cancer will not receive the specialised service they need. Furthermore, GPs and RNs in primary care were often not supplied with information from patient’s medical records before all treatment had ended in specialised care, at which point patients with lung cancer were transferred home for end-of-life care. Consequently, a central part of the RNs’ understanding of home care for patients with lung cancer concerns palliative and end-of-life care.

According to the World Health Organization [33], the core value of palliative care is improving the quality of life of patients and their relatives, indicating that the specific diagnosis is not the focus. Also central to palliative care is a focus on enabling patients to live at home for as long as possible. When doing so, HCPs, like those in our study, must consider several different aspects of home care, such as a threshold or other physical obstacles hindering the patient from entering the bathroom, the level of involvement of the patient’s relatives, or whether they have the medical support available when the patient’s medical condition worsens. The participants in our study expressed frustration over how HCPs in specialised care were rarely concerned about this, as they often failed to communicate and prioritise information in relation to patients’ care at home. Such a contrast in what we describe as differing logics of care is supported by Martens and Veenstra [34], who concluded that HCPs in specialised care must improve their competence concerning primary care services. Confirming those findings, our participants also expressed that HCPs in specialised care need knowledge of primary care organisation and practice in order to make the exchange of information more relevant for HCPs in primary care.

Furthermore, our findings indicate that the reduced practice of physical meetings between HCPs in specialised and primary care also reduces opportunities to openly discuss one another’s challenges and needs on common ground. While electronic messages have made communication more efficient between the two levels of healthcare services [35], our findings indicate that reliance on electronic messaging alienates HCPs in specialised and primary care, making cooperation to ensure a good transition more challenging. We argue that this alienation of their respective practices exacerbates the differences in the logic of care between HCPs in specialised and primary care. Gautun et al. [20] support this argument, concluding that RNs working with older adults in hospitals and communities (nursing homes and home care) should have more face-to-face or telephone contact to improve hospital-to-home transitions. Removing the opportunity to have physical meetings to discuss the patient’s needs and challenges also removes the opportunity for informal contact. Therefore, this mode of communication appears to be contrary to the ‘logic of care’ among HCPs in primary care. As our participants described, the first thing they did when receiving a new patient was to get to know them through an initial conversation. While they usually received the discharge and nursing summary from specialised care, they still felt the need to hold a face-to-face conversation with the patient to obtain sufficient information to adequately care for them at home.

### Differences in prestige and influence

Supported by previous research [20, 36–38], our findings are that HCPs in primary and specialised care lack sufficient understanding of each other’s practices. While both groups operate within the medical field and have their patients’ best interests in mind, they exhibit considerable differences in their respective understandings of how those interests are best served in practice. Our findings indicate that such differences are also connected to imbalances in influence and resources, largely to the detriment of HCPs in primary care. As mentioned above, RNs and GPs in our study called for deeper involvement from HCPs in specialised care and a more relevant information flow before discharge or after treatment at the outpatient clinic. This information exchange was often a one-way communication from HCPs in specialised care. While the RNs providing home-based care had relevant information after caring for the patient between visits to the outpatient clinic, they lacked a digital platform to share their information with HCPs in specialised care, which can be seen as a failure to acknowledge not only the ways HCPs in primary care conduct their work, but also their expertise.

Previous studies have found that HCPs rank different medical specialities and diseases according to a hierarchy of prestige [39–42]. Medical specialities that commonly fall under specialised care, such as neurosurgery and thoracic surgery, are ranked high, whereas general medical practice is ranked low. Similarly, acute diseases with a high probability of fatal outcomes or advanced surgery are ranked high, whereas chronic diseases with an extended treatment period are ranked low; the more advanced or acute a disease, the greater its prestige. Thus, the more ‘prestigious’ diseases belong to specialised care, whereas lower-ranked diseases belong to primary care. In other words, HCPs working in specialised care occupy a dominant position within the medical field, articulated through the prestigious diseases they treat and the medical specialities they represent [41]. Prestige also shapes a professional hierarchy among RNs and physicians, which is evident in how HCPs can provide and obtain patient information. For instance, GPs and hospital physicians can exchange information digitally when patients receive treatment at the outpatient clinic, unlike RNs, who cannot.

The manner in which status and prestige divide specialised and primary care can also be seen in how work tasks are transferred from specialised care to primary care. Task shifting is a well-known tactic in healthcare for optimising the use of available health resources, often by delegating specific tasks from highly skilled HCPs to those with less training and fewer qualifications [43]. In Norway, the Coordination Reform [12] was also designed to transfer tasks from specialised to primary care in the same way, specifically by focusing on shorter hospital stays and more advanced services being provided in the primary care setting. The implementation of the Coordination Reform marked a continuation of the governing principles in Norwegian health policies, advocating that all healthcare should be conducted at the lowest, most effective care level [44].

In the context of our study, the transfer of work tasks from specialised to primary care assigns the latter not only more tasks but also more prestigious ones. However, it is crucial to note that without the necessary training and resources, shifting tasks primarily benefits HCPs in specialised care, who are released from some of their tasks and responsibilities. Therefore, proper training and economic resources are urgently needed to ensure the equitable distribution of tasks. The fact that HCPs in primary care inherit tasks formerly undertaken by HCPs in specialised care that they are ill-equipped to perform could adversely affect patients’ experiences of hospital-to-home transitions and, ultimately, endanger their safety. This issue is also illustrated in our findings, where the RNs explained how they lack medical support to care for critically ill patients in their homes or how GPs lack the resources to perform home visits to terminally ill patients. Our findings illuminate how primary care holds less influence within the medical field, representing a practice with lower prestige than specialised care. In our context, specialised care can define where their responsibility starts and ends. In contrast, HCPs in primary care must embrace the responsibility of caring for patients at home, in addition to the tasks inherited from specialised care [45].

### Strengths and limitations

Our study had several limitations. Firstly, its sample size was limited. However, we found that we had sufficient data to answer the research question. Secondly, the researchers had no way of knowing how many participants were invited by the gatekeeper compared to how many were recruited. Thirdly, there were more females than males among the participating RNs. Fourthly, the participating GPs may have a particular interest in transitions, performing home visits, and cooperating well with RNs providing home-based care and thus may not generally be representative of all GPs. Fifthly, the financial and other resources of the examined municipalities vary, which might have affected the representativeness of our study sample. Sixthly, the use of focus group interviews entails certain limitations. For example, some participants may feel intimidated by more experienced and dominant group members; if they are hesitant to share their experiences, that will reduce the generalisability of the results. Seventhly, the initially planned focus group interviews with GPs were not conducted and were replaced with dyadic and individual interviews. However, the dyadic interviews also allowed us to observe the interaction dynamics between the participants. In contrast, the individual interview offered a more detailed and profound understanding of the participant’s experience. It was essential to gain insight into GPs’ experience as they play a vital role in hospital-to-home transitions and are considered a cooperation partner for RNs in in-home care. Therefore, adapting the initially planned focus group interviews with GPs into two dyadic interviews and one individual interview was accepted as a potential limitation to include insights into GPs’ experiences compared to not having any interviews with GPs. Nonetheless, one strength of our study was that it interviewed RNs providing home care and GPs to obtain their experiences in hospital-to-home transitions, enabling us to explore challenges within primary care, between the two professions, and across different service levels. Eighthly, the first author (who conducted the focus group interviews) was a PhD candidate with no prior experience conducting focus group interviews. Nonetheless, all co-moderators were experienced senior researchers with substantial previous experience with that type of interview. Finally, analysing the dataset as a whole could have impeded our ability to identify variations, contradictions, or unique contributions from each source. However, our results consist of rich quotes from the interviews, each marked with a code indicating the specific interview from which the quotes were taken.

Because the focus group interviews were homogenously classed by profession, all group members worked together and knew one another, promoting a relaxed group dynamic [46]. Focus group interviews were chosen because they are suitable for gaining insights into organisational issues and understanding employees’ concerns. They also give participants more opportunities to reflect on, listen to, and consider the group’s experience with the topics discussed. Moreover, they facilitate reflection on current practices. Dyadic and individual interviews were chosen for practical reasons. Finding time to gather GPs for a focus group interview was challenging since the GP offices were far apart. In addition, due to infection control measures during the COVID-19 pandemic, the gathering of HCPs from different health institutions in one location was restricted.

## Conclusions

In conclusion, our findings indicate that HCPs in specialised and primary care follow different logics of care, leading them to prioritise differentially – and thus challenge – the policies and goals of hospital-to-home transitions. In the medical field, encompassing both specialised and primary care, our findings address how primary care has less power to define how hospital-to-home transitions should be practised. Our participants highlighted a different focus on how to care for the patient at home, e.g. how to live with the challenges the disease brings. However, HCPs in specialised care hold the power to define and prioritise because, in this case, they are the experts on lung cancer, possessing specialised competence in care required by patients with lung cancer. This imbalance of agency and authority manifests as opposing logics of care in which diagnosing and treating lung cancer emerge as the dominant clinical practice, contrasting with a logic that emphasises the quality of life of patients with lung cancer.

Our findings support the argument that primary care must receive increased resources to adequately meet its increased medical responsibilities following the Norwegian Coordination Reform and that the existing digital platforms for collaboration must be adapted to current clinical practices. Specifically, our findings highlight a lack of medical support for RNs providing home-based care, delays and deficiencies in information exchange, and insufficient time and space for communication and collaboration to ensure adequate hospital-to-transitions for vulnerable patients, such as those with lung cancer. We propose that closer collaboration and more frequent face-to-face meetings may reduce the need for RNs and GPs to search for relevant information after hospital-to-home transitions. The future goal is a more seamless transition experience in which all participating HCPs are fully aware of the patient’s situation.

## Data Availability

The data that support the findings of this study are available from the corresponding author upon reasonable request.
